# A quantitative analysis of postural control in elderly patients with vestibular disorders using visual stimulation by virtual reality^☆^

**DOI:** 10.1016/j.bjorl.2019.03.001

**Published:** 2019-04-22

**Authors:** Juliana Maria Gazzola, Heloísa Helena Caovilla, Flávia Doná, Maurício Malavasi Ganança, Fernando Freitas Ganança

**Affiliations:** a Universidade Federal de São Paulo, Departamento de Otorrinolaringologia e de Cirurgia de Cabeça e Pescoço, São Paulo, SP, Brazil; b Universidade Federal do Rio Grande do Norte, Departamento de Fisioterapia, Natal, RN, Brazil

**Keywords:** Aging, Accidental falls, Postural balance, Vestibular diseases, Envelhecimento, Quedas acidentais, Equilíbrio postural, Doenças vestibulares

## Abstract

**Introduction:**

Postural instability is one the most common disabling features in vestibular disorders.

**Objective:**

This study aimed to analyze the limit of stability and the influence of manipulation of visual, somatosensorial and visual–vestibular information on postural control in older adults with vestibular disorder, with and without a history of falls.

**Methods:**

Cross-sectional study. Participants – 76 elderly patients with vestibular disorder (G1, without falls; G2, with falls) and 41 healthy elderly subjects (control group; CG). Using posturography, analyzed were limit of stability area, body center of pressure, and velocity of oscillation in the standing position in 10 conditions, including open/closed eyes, unstable surface with eyes closed, saccadic and optokinetic stimuli, and visual–vestibular interaction.

**Results:**

Limit of stability area in CG was better compared with G1-2, and center of pressure values were worse in G1 than in CG. Center of pressure area in all conditions and velocity of oscillation in the following conditions: open/closed eyes, optokinetic stimulation, and visual–vestibular interaction showed worse values in G2 than in CG. Center of pressure area in the following conditions: open/closed eyes, saccadic and optokinetic stimuli, visual–vestibular interaction, and unstable surface with eyes closed showed worse values in G2 than in G1.

**Conclusion:**

Older adults with vestibular disorder presented reduced limit of stability and increased postural sway in the following conditions: conflict between visual and somatosensory information and visual–vestibular interaction. Deterioration in postural control was significantly associated with history of falls.

## Resumo

**Introdução:**

Instabilidade postural é uma das características incapacitantes mais comuns nos distúrbios vestibulares.

**Objetivo:**

Analisar o limite de estabilidade e a influência da manipulação de informações visuais, somatossensoriais e visuais-vestibulares no controle postural em idosos com disfunção vestibular, com e sem histórico de quedas.

**Método:**

Estudo transversal. Participantes: 76 idosos com distúrbios vestibulares (G1, sem quedas; G2, com quedas) e 41 idosos saudáveis (grupo controle; GC). Com o uso da posturografia, foram analisadas a área do limite de estabilidade, centro de pressão e velocidade de oscilação na posição ereta em 10 condições, incluindo olhos abertos/fechados, superfície instável com olhos fechados, estímulos sacádicos e optocinéticos e interação visual-vestibular.

**Resultados:**

A área de limite de estabilidade no GC foi melhor comparada com o G1-2 e os valores do centro de pressão foram piores no G1 do que no GC. A área do centro de pressão em todas as condições e a velocidade de oscilação nas seguintes condições: olhos abertos/fechados, estímulo optocinético e interação visual-vestibular mostraram valores piores no G2 do que no GC. A área do centro de pressão nas seguintes condições: olhos abertos/fechados, estímulos sacádicos e optocinéticos, interação visual-vestibular e superfície instável com olhos fechados apresentou valores piores no G2 do que no G1.

**Conclusão:**

Idosos com disfunção vestibular apresentaram redução de limite de estabilidade e aumento da oscilação postural nas seguintes condições: conflito entre informação visual e somatossensorial e interação visual-vestibular. A deterioração no controle postural foi significantemente associada ao histórico de quedas.

## Introduction

Postural instability or decreased body balance is one of the main causes of handicap among the elderly^1^, ^2^ as the final outcome may be the occurrence of falls.^3^, ^4^ A vestibular disorder may affect body balance, and the prevalence of falls is greater in this population (53.3%)^5^ than in community elderly (32.7–34.8%).^6^, ^7^ The most prevalent causes of postural instability complaint, dizziness, and/or falls are benign paroxysmal positional vertigo, Ménière's disease, vestibular hypofunction (unilateral or bilateral), vascular labyrinth disorder, metabolic labyrinth disorder, and neurological problems, such as Parkinson's disease, stroke, and multiple sclerosis.^8^, ^9^

Posturographic assessment may be useful in the analysis of body instability of elderly individuals^10^, ^11^, ^12^ and in the identification of risk of falls.^13^, ^14^, ^15^ Results of posturographic tests should be evaluated with other information obtained in the propaedeutic of patients with vestibular disorder, such as clinical history, physical examination, laboratory tests, and functional examinations of body balance.^16^, ^17^

Virtual reality systems integrated to a force platform have been used for visual stimulation and to assess the effect of sensorial perception on postural responses when stimuli are provided. Virtual reality is an “advanced interface” for computational applications, which is characterized by visualization and movement in tridimensional environments in real time as well as interaction with elements from this environment.^11^, ^12^

Identifying the posturographic performance of elderly individuals with chronic vestibular disorder in different visual environments that stimulate oculomotor reflexes, such as saccadic and optokinetic, pursuing and visual–vestibular interaction may be useful in determining visual and vestibular stimuli that result in greater instability and in designing rehabilitation protocols of body balance, especially for those with a greater risk of falling. Using posturography integrated to virtual reality, this study aimed to evaluate postural control of elderly individuals with chronic vestibular disorder with or without falls

## Methods

### Subject selection

A cross-sectional study was performed from December 2007 to March 2010, approved by the Research Ethics Committee of the Federal University of São Paulo (UNIFESP). The work described has been carried out in accordance with The Code of Ethics of the World Medical Association (protocol number 1704/07). Elderly male and female individuals aged ≥ 65 years were assessed and assigned into the Control Group (CG) and group of patients with chronic vestibular disorder, which was subdivided into two groups: Group 1 (G1) – patients without a history of falls in the past 6 months and Group 2 (G2) – patients with a history of falls within the same period.

Exclusion criteria for all three groups were as follows: elderly individuals unable to understand and to respond to simple verbal commands; those unable to stay independently in the orthostatic position and who use an auxiliary device for gait; those with severe visual impairment without corrective lenses; those with diabetes and on insulin therapy with an orthopedic disorder resulting in movement limitation and use of prosthesis in the lower limbs; those with neurological and/or psychiatric disorders; those who had ingested alcohol 24 h before the evaluation; those receiving medications with action on the central nervous system or vestibular system; and those who underwent body balance rehabilitation in the past 6 months.

CG was composed of elderly patients from the Aging Studies Sector at UNIFESP. Inclusion criteria for CG were the following: absence of vestibular and/or unbalance symptoms, without hearing complaints, without personal background of vestibular disease, without abnormal signs in the assessment of vestibular function, without a history of falls in the past 6 months, and with difficulty in at most three activities of daily living verified by the Brazilian Older Americans Research and Services Multidimensional Functional Assessment Questionnaire (BOMFAQ).^18^

### Clinical protocol

The diagnosis of chronic vestibular disorder was considered when vestibular dizziness was present for at least three consecutive months.^19^ All study participants provided information on gender, age, height, regular physical activity, number of diseases, and number of medications and answered the BOMFAQ questionnaire.^18^ Their vestibular function was assessed, and electronystagmography and static posturography integrated to virtual reality was performed.^9^, ^20^

Physical activity was considered regular when the participant reported performing it three or more times a week, during their free time, for >30 min in the last 14 days.^21^

### Computerized posturography

The Balance Rehabilitation Unit (BRU; Medicaa, Montevideo, Uruguay) is a computerized posturography system integrated with a virtual reality system that measures postural sway resulting from different stimuli.^9^, ^12^, ^20^ It uses information on the position of the subject's Center of Pressure (COP) during tasks performed on a balance platform (450 × 450 mm). The BRU software sends a stimulus to a head-mounted display (eMagin Z800 3D Vision, New York, NY, USA), which elicits oculomotor reflexes (saccadic, optokinetic, and vestibulo-ocular).

The parameters analyzed were Limit Of Stability (LOS) and 95% Confidence Intervals of COP, and the mean Velocity of Oscillation (VOS) was determined by the total COP trajectory divided by the test time (60 s).^9^ The LOS area was quantified by the total maximum and minimum displacement in the Y-axis (anteroposterior) and *X*-axis (midlateral), approaching the oscillation pattern to an ellipse. The LOS test was employed to evaluate the ability to displace one's COP in the anteroposterior and lateral planes without risk of falling. An increase in LOS indicates good stability. COP represents the ground reaction force vector and reflects movement of the body's center of mass. Anteroposterior and midlateral displacements were recorded at a sampling frequency of 50 Hz. COP displacements were used to estimate COP sway area.

Posturography assessment was performed with the patient in the orthostatic position on the platform, arms alongside the body, and barefoot with the internal malleolus positioned on the extremities of the intermalleolar line.

To determine the LOS, participants were asked to shift their body to anteroposterior and midlateral directions through the ankle strategy, that is, without moving the feet or using trunk strategies, for 60 s. The procedure was repeated up to three times when the participants moved their feet or trunk.

The participants were assessed in the orthostatic position in 10 sensorial conditions classified according to visual stimulus and to the involved oculomotor reflex^12^, ^20^: Condition 1 (firm surface, open eyes); Condition 2 (firm surface, closed eyes); Condition 3 (unstable foam surface, closed eyes); Condition 4 (firm surface, saccadic stimulation at the frequency of 0.2 KHz); Condition 5 (firm surface, horizontal optokinetic stimulation from left to right); Condition 6 (firm surface, horizontal optokinetic stimulation from right to left); Condition 7 (firm surface, vertical up-down optokinetic stimulation); Condition 8 (firm surface, vertical down-up optokinetic stimulation); Condition 9 (firm surface, horizontal optokinetic stimulation associated with slow and uniform head rotation movements with firm shoulders and trunk) and Condition 10 (firm surface, vertical optokinetic stimulation associated with slow and uniform flexo-extension head movement with firm shoulders and trunk). In Conditions 5–8, the angular velocity of the optokinetic stimulation was 60°/s.

Virtual reality goggles (eMagin Z800 3D Vision, eMagin, New York) provided visual stimuli to trigger oculomotor reflexes (saccadic and optokinetic), pursuing, and visual–vestibular interaction, that is, optokinetic stimulation associated with Vestibulo-Ocular Reflex (VOR) by vertical and horizontal head movements. The virtual reality goggles were used from the fourth to the tenth sensorial conditions. In Condition 4, the subject was instructed to follow randomized visual targets with different colors and letters to stimulate the saccadic system. The visual stimulus displayed in the subject's visual field in Conditions 5–8 had moving individual black bars. The subject was instructed to look at the center of the visual field to induce optokinetic nystagmus.

For the assessment of each of the ten conditions, the participants were asked to stay in the orthostatic position (static and silent and as calm as possible) for 60 s.

A 14 cm high-density polyurethane foam was used in the third condition; a protection support system with harnesses and a safety belt were used to avoid falls. Inability to maintain body balance was described as the use of the step strategy or movement of the upper limbs, calcaneus, or feet to compensate for body instability after the third repetition of the procedure in each condition.

### Statistical analysis

Simple descriptive and bivariate statistical analyses were performed using chi-square test or Fisher's test and ANOVA (followed by Bonferroni test for multiple comparison, when *p* < 0.05). The significance level was set at 5% (*α* = 0.05). Statistical Package for Social Sciences (SPSS, version 10.0, 1999) for Windows was used for the statistical analysis. The sample size estimated was based on previously collected posturography data with the following assumption: *α* = 0.05 and 0.80 power analysis.

## Results

### Clinical characteristics

A total of 117 elderly individuals participated in this study (chronic vestibular disorder, 76; healthy ones, 41). Elderly individuals in G2 had at least one episode of fall: 23 (19.7%) fell only once and 13 (11.3%) reported recurrent episodes of falls in the past 6 months.

The descriptive analysis of age, height, number of diseases, number of medications, and functional capacity of each group and the comparative analysis between CG and G1, G1 and G2, CG and G2 are presented in Table 1.

**Table 1:** Descriptive and comparative analyses of age, gender, height, number of diseases, number of medications, functional capacity, and physical activity practice between elderly individuals in the control group and elderly individuals with chronic vestibular disorder with and without a history of falls.

	Groups	Average (SD)	Minimum and maximum values	Comparative analysis (CG-G1-G2)
Age				CG-G1-G2 (*p* = 0.11)^a^
Average (SD)/Variation	CG (*n* = 41)	72.51 (6.84)	65–85	
	G1 (*n* = 40)	71.90 (5.23)	65–85	
	G2 (*n* = 36)	73.92 (6.27)	65–85	
Height (meters)				CG-G1-G2 (*p* = 0.16)^a^
Average (SD)/Variation	CG (*n* = 41)	1.61 (0.07)	1.47–1.78	
	G1 (*n* = 40)	1.58 (0.08)	1.40–1.75	
	G2 (*n* = 36)	1.57 (0.08)	1.45–1.78	
Number of diseases				CG-G1-G2 (*p* < 0.001)^a^
Average (SD)/Variation	CG (*n* = 41)	1.88 (1.26)	0–6	G1-CG (*p* < 0.001)^b^
	G1 (*n* = 40)	3.25 (1.48)	0–6	G2-CG (*p* < 0.001)^b^
	G2 (*n* = 36)	3.38 (1.61)	1–8	G1-G2 (*p* = 1.000)^b^
Number of medications				CG-G1-G2 (*p* < 0.001)^a^
Average (SD)/Variation	CG (*n* = 41)	1.63 (1.29)	0–5	G1-CG (*p* < 0.001)^b^
	G1 (*n* = 40)	3.88 (2.11)	0–10	G2-CG (*p* < 0.001)^b^
	G2 (*n* = 36)	4.17 (2.31)	0–9	G1-G2 (*p* = 1.000)^b^
Functional capacity BOMFAQ				CG-G1-G2 (*p* < 0.001)^a^
Average (SD)/Variation	CG (*n* = 41)	1.00 (1.05)	0–3	G1-CG (*p* < 0.001)^b^
	G1 (*n* = 40)	4.53 (2.61)	0–13	G2-CG (*p* < 0.001)^b^
	G2 (*n* = 36)	5.42 (3.69)	0–3	G1-G2 (*p* = 0.43)^b^
Gender, *n* (%)		Female	Male	CG-G1-G2 (*p* = 0.28)^c^
	CG (*n* = 41)	24 (30.4%)	17 (44.7%)	
	G1 (*n* = 40)	28 (35.4%)	12 (31.6%)	
	G2 (*n* = 36)	27 (34.2%)	9 (23.7%)	
Physical activity, *n* (%)		Yes	No	CG -G1-G2 (*p* = 0.002)^c^
	CG (*n* = 41)	24 (51.1%)	17 (24.3%)	G1-CG (*p* = 0.10)^c^
	G1 (*n* = 40)	16 (34.0%)	24 (34.3%)	G2-CG (*p* < 0.001)^c^
	G2 (*n* = 36)	7 (14.9%)	29 (41.4%)	G1-G2 (*p* = 0.05)^c^

CG, Control Group; G1, without falls; G2, with falls; SD, Standard Deviation; BOMFAQ, Brazilian OARS (Older Americans Research and Services) Multidimensional Functional Assessment Questionnaire [16]. Significance level *α* = 0.05.

a ANOVA.

b Bonferroni test.

c Chi-square.

### Posturography analysis

Of the 76 elderly individuals with chronic vestibular disorder, four women aged 72–83 years in G2 did not perform all conditions: three did not complete Condition 3 and one did not complete Conditions 2 and 3.

The value of LOS was significantly higher in CG than in G1, and no significant difference was found between G1 and G2 (Table 2).

**Table 2:** Descriptive and comparative analyses of stability limit area (cm^2^) and COP area (cm^2^) in the conditions tested between elderly individuals in the control group and elderly individuals with chronic vestibular disorder with and without a history of falls.

	Groups	Average (SD)	Minimum and maximum values	ANOVA/Bonferroni test (CG-G1-G2)
Stability limit area (cm^2^)				*p* = 0.013^a^
	CG (*n* = 41)	154.71 (55.10)	137.31–172.10	G1-CG (*p* = 0.038)^b^
	G1 (*n* = 40)	125.78 (51.05)	109.45–142.10	G2-CG (*p* = 0.024)^b^
	G2 (*n* = 36)	126.53 (49.44)	108.71–144.36	G1-G2 (*p* = 0.97)^b^
COP area (cm^2^) of conditions				
FS/Open eyes/Without stimulus				*p* < 0.001^a^
	CG (*n* = 41)	1.86 (1.05)	1.53–2.19	G1-CG (*p* = 0.15)^b^
	G1 (*n* = 40)	2.48 (1.35)	2.05–2.90	G2-CG (*p* < 0.001)^b^
	G2 (*n* = 36)	5.16 (4.90)	3.35–6.29	G1-G2 (*p* = 0.002)^b^
FS/Closed eyes				*p* < 0.001^a^
	CG (*n* = 41)	1.77 (0.98)	1.45–2.08	G1-CG (*p* = 0.015)^b^
	G1 (*n* = 40)	3.03 (2.23)	2.31–3.75	G2-CG (*p* < 0.001)^b^
	G2 (*n* = 35)	6.69 (7.83)	3.85–9.73	G1-G2 (*p* = 0.003)^b^
Foam/Closed eyes				*p* < 0.001^a^
	CG (*n* = 41)	10.59 (7.94)	8.08–13.09	G1-CG (*p* = 0.20)^b^
	G1 (*n* = 40)	12.61 (7.03)	10.36–14.86	G2-CG (*p* < 0.001)^b^
	G2 (*n* = 32)	20.94 (13.73)	15.99–25.89	G1-G2 (*p* = 0.014)^b^
FS/Saccadic				*p* = 0.003^a^
	CG (*n* = 41)	2.16 (0.99)	1.84–2.46	G1-CG (*p* = 1.000)^b^
	G1 (*n* = 40)	2.42 (1.32)	1.99–2.84	G2-CG (*p* = 0.002)^b^
	G2 (*n* = 36)	3.90 (3.06)	2.68–4.83	G1-G2 (*p* = 0.040)^b^
FS/Bars/Right optokinetic				*p* = 0.010^a^
	CG (*n* = 41)	2.09 (1.03)	1.76–2.41	G1-CG (*p* = 0.35)^b^
	G1 (*n* = 40)	2.71 (1.82)	2.13–3.29	G2-CG (*p* = 0.007)^b^
	G2 (*n* = 36)	3.86 (3.04)	2.80–5.03	G1-G2 (*p* = 0.38)^b^
FS/Bars/Left optokinetic				*p* = 0.002^a^
	CG (*n* = 41)	2.27 (1.29)	1.86–2.67	G1-CG (*p* = 1.000)^b^
	G1 (*n* = 40)	2.60 (2.64)	1.75–3.44	G2-CG (*p* = 0.003)^b^
	G2 (*n* = 36)	4.07 (3.12)	2.78–4.88	G1-G2 (*p* = 0.019)^b^
FS/Bars/Downward optokinetic				*p* < 0.001^a^
	CG (*n* = 41)	2.06 (1.13)	1.69–2.41	G1-CG (*p* = 0.22)^b^
	G1 (*n* = 40)	2.93 (2.29)	2.12–3.66	G2-CG (*p* < 0.001)^b^
	G2 (*n* = 36)	4.01 (2.47)	2.99–4.72	G1-G2 (*p* = 0.06)^b^
FS/Bars/Upward optokinetic				*p* = 0.001^a^
	CG (*n* = 41)	2.30 (1.21)	1.91–2.68	G1-CG (*p* = 1.000)^b^
	G1 (*n* = 40)	2.86 (2.48)	2.06–3.65	G2-CG (*p* = 0.001)^b^
	G2 (*n* = 36)	4.98 (4.54)	3.39–6.33	G1-G2 (*p* = 0.017)^b^
SF/Bars/VVI/Horizontal				*p* < 0.001^a^
	CG (*n* = 41)	3.17 (1.83)	2.59–3.74	G1- CG (*p* = 0.018)^b^
	G1 (*n* = 40)	4.65 (3.10)	3.66–5.64	G2-CG (*p* < 0.001)^b^
	G2 (*n* = 36)	7.24 (5.09)	5.52–8.96	G1-G2 (*p* = 0.018)^b^
SF/Bars/VVI Vertical				*p* = 0.029^a^
	CG (*n* = 41)	3.62 (1.83)	3.04–4.19	G1-CG (*p* = 0.68)^b^
	G1 (*n* = 40)	4.63 (3.89)	3.38–5.87	G2-CG (*p* = 0.024)^b^
	G2 (*n* = 36)	5.60 (4.11)	4.20–6.98	G1-G2 (*p* = 0.41)^b^

SD, Standard Deviation; CG, Control Group; G1, without falls; G2, with falls; FS, Firm Surface; VVI, Visual–Vestibular Interaction. Significance level *α* = 0.05.

a ANOVA.

b Bonferroni test.

The COP area values were significantly different between the groups in all conditions, and the values were higher in G2 than in CG. COP area values in G1 were higher than those in CG in Conditions 2 and 9. Significant differences were also found between G1 and G2 in Conditions 1–4, 6, 8, and 9.

The VOS values were significantly different between the groups in Conditions 1, 2, 7, 8 and 9, with values higher in G2 than in CG. In addition, significant differences were found between G1 and G2 in Conditions 1 and 2 (Table 3).

**Table 3:** Descriptive and comparative analyses of velocity of oscillation (cm/s) in the conditions tested between elderly individuals in the control group and elderly individuals with chronic vestibular disorder with and without a history of falls.

Velocity of oscillation (cm/s) of conditions	Groups	Average (SD)	Minimum and maximum values	ANOVA/Bonferroni test (CG-G1-G2)
FS/Open eyes/Without stimulus				*p* = 0.002^a^
	CG (*n* = 41)	0.89 (0.26)	0.80–0.96	G1-CG (*p* = 1.000)^b^
	G1 (*n* = 40)	0.92 (0.25)	0.83–0.99	G2-CG (*p* < 0.001)^b^
	G2 (*n* = 36)	1.17 (0.46)	0.99–1.28	G1-G2 (*p* = 0.03)^b^
FS/Closed eyes				*p* = 0.001^a^
	CG (*n* = 41)	1.11 (0.41)	0.98–1.23	G1-CG (*p* = 0.59)^b^
	G1 (*n* = 40)	1.23 (0.43)	1.09–1.36	G2-CG (*p* < 0.001)^b^
	G2 (*n* = 35)	1.59 (0.67)	1.34–1.84	G1-G2 (*p* = 0.03)^b^
Foam/Closed eyes	CG (*n* = 41)	2.87 (1.36)	2.44–3.30	*p* = 0.05^a^
	G1 (*n* = 40)	3.06 (1.11)	2.71–3.41	
	G2 (*n* = 32)	3.45 (1.15)	3.04–3.86	
FS/Saccadic	CG (*n* = 41)	1.25 (0.63)	1.05–1.45	*p* = 0.14^a^
	G1 (*n* = 40)	1.35 (0.48)	1.20–1.50	
	G2 (*n* = 36)	1.46 (0.59)	1.23–1.68	
FS/Bars/Right optokinetic	CG (*n* = 41)	1.25 (0.55)	1.07–1.42	*p* = 0.42^a^
	G1 (*n* = 40)	1.23 (0.39)	1.10–1.35	
	G2 (*n* = 36)	1.36 (0.49)	1.19–1.55	
FS/Bars/Left optokinetic	CG (*n* = 41)	1.19 (0.52)	1.02–1.35	*p* = 0.24^a^
	G1 (*n* = 40)	1.17 (0.42)	1.03–1.30	
	G2 (*n* = 36)	1.33 (0.47)	1.14–1.48	
FS/Bars/Downward optokinetic				*p* = 0.030^a^
	CG (*n* = 41)	1.14 (0.47)	0.98–1.28	G1-CG (*p* = 1.000)^b^
	G1 (*n* = 40)	1.21 (0.47)	1.06–1.36	G2-CG (*p* = 0.03)^b^
	G2 (*n* = 36)	1.41 (0.54)	1.21–1.60	G1-G2 (*p* = 0.24)^b^
FS/Bars/Upward optokinetic				*p* = 0.014^a^
	CG (*n* = 41)	1.14 (0.46)	0.99–1.28	G1-CG (*p* = 1.000)^b^
	G1 (*n* = 40)	1.18 (0.40)	1.05–1.30	G2-CG (*p* = 0.015)^b^
	G2 (*n* = 36)	1.50 (0.72)	1.27–1.73	G1-G2 (*p* = 0.08)^b^
SF/Bars/VVI/Horizontal				*p* = 0.034^a^
	CG (*n* = 41)	1.47 (0.65)	1.26–1.67	G1-CG (*p* = 0.49)^b^
	G1 (*n* = 40)	1.64 (0.66)	1.42–1.85	G2-CG (*p* = 0.03)^b^
	G2 (*n* = 36)	1.86 (0.83)	1.61–2.22	G1-G2 (*p* = 0.63)^b^
SF/Bars/VVI/Vertical	CG (*n* = 41)	1.69 (0.69)	1.47–1.90	*p* = 0.62^a^
	G1 (*n* = 40)	1.67 (0.54)	1.49–1.83	
	G2 (*n* = 36)	1.79 (0.60)	1.61–2.04	

SD, Standard Deviation; CG, Control Group; G1, without falls; G2, with falls; FS, Firm Surface; VVI, Visual–Vestibular Interaction. Significance level *α* = 0.05.

a ANOVA.

b Bonferroni test.

## Discussion

The mean age of the elderly in this study was 72 years, which is similar to the mean age of other researches that assessed elderly individuals with dizziness and/or body imbalance.^5^, ^10^, ^11^, ^22^, ^23^ The prevalence of vestibular system disorders in women was also documented,^5^, ^24^ which corroborated the findings of our study.

The diagnosis of vestibular disorder, the number of associated vestibular affections, and the onset, duration, and periodicity of dizziness, functional capacity, and fear of falling were similar between groups with and without falls; hence, these groups are clinically similar, and the occurrence of falls is the main difference. However, the association between rotatory and non-rotatory dizziness was prevalent in G2; thus, this group possibly has more than one disease^24^ or a concomitant damage in other systems involved in body balance, thereby suggesting a multifactorial sensorial disorder.

Four cases in G2 presented difficulty in completing the posturographic assessment in Conditions 2 (firm surface and closed eyes) and/or 3 (foam surface and closed eyes) because of the inability to maintain the orthostatic position. Nevertheless, elderly individuals with recurrent falls had a significantly higher loss of body balance in the third attempt of the test than those without falls, and those who fell only once, which suggested that loss of body balance during posturography could be associated with the risk of falls during activities of daily living.^25^

LOS was significantly higher in CG than in G1 and G2, which is similar to other studies using the same posturography equipment.^11^ LOS is the area where the patient's oscillation is safer; a significant reduction in this area may result in increased risk of falling as subtle COP oscillation exceeds LOS.^9^, ^11^, ^20^ These concepts may justify why elderly individuals with body balance disorders due to fear of falling, did not reach their real limits during the test and presented reduction in LOS compared with healthy individuals. The worse performance of G1 and G2 regarding LOS may be justified by a dysfunction of the vestibular and other systems involved in body balance.

In Condition 1 (firm surface and open eyes), differences in the COP area and VOS between G2 and CG and between G1 and G2 were observed, revealing higher values in G2 where body balance was worse. This finding was also reported in other studies.^11^, ^22^, ^26^ However, no significant difference in these parameters between CG and G1 were found, which is consistent with the finding that the use of visual and somatosensory clues could compensate the imprecise information of the vestibular system for the maintenance of body balance in patients with vestibular disorder.^10^

In Condition 2 (firm surface and closed eyes), differences in the COP area between the three groups and differences in the VOS between G2 and CG and between G1 and G2 were found, which emphasized that visual input has a significant role in body balance, especially in elderly individuals with somatosensory disorders due to the aging process^27^ and/or a history of falls.^4^, ^28^ Similar findings were reported between the following groups of healthy and functionally active elderly individuals: never fell, a history of one fall, and a history of two or more falls, based on the score of balance for Condition 2 in the Sensory Organization Test (firm surface and closed eyes)^15^, ^25^, ^28^ and in the condition of Romberg with closed eyes.^22^ VOS did not differ between G1 and CG, corroborating the findings of other studies.^22^, ^26^

In Condition 3 (foam surface and closed eyes), where somatosensory information is imprecise with a foam surface and visual information is absent, the vestibular system acts as the main source of sensorial information for postural control. Differences in the COP area between G2 and CG and between G1 and G2 were observed, demonstrating a worse performance of elderly individuals with vestibular disorder with a history of falls. VOS did not differ between the groups in this condition. Previous studies reported that in Condition 3, no significant differences in VOS between elderly individuals with balance disorders and/or dizziness and those in CG or between elderly individuals who reported falls and those who did not were observed.^26^, ^28^ These findings may be attributed to postural fixation and the fear of falling during the test.^29^ The third condition is clearly the most challenging for postural control; values of COP area and VOS were greater in this condition than in other conditions. The decreased somatosensory information, especially with closed eyes, increases vestibular function dependency, which in turn elevates the risk of falling in patients with vestibular disorders.

In Condition 4 (firm surface and saccadic stimulus), some differences in the COP area were noted between G2 and CG and between G1 and G2, with higher values in G2. This finding was also reported in a previous study.^11^ VOS did not differ between the groups in Condition 4, which is consistent with the finding of previous study,^30^ but not with another study.^11^

In Condition 5 (firm surface and right optokinetic stimulation), a significant difference in the COP area only between G2 and CG was found. In Condition 6 (firm surface and left optokinetic stimulation), a significant difference in the COP area between G2 and CG and between G2 and G1 was noted. In Conditions 7 and 8 (firm surface and vertical up-down; down-up optokinetic stimulation), the COP area and VOS were significantly different between G2 and CG. These findings suggest that vertical optokinetic stimulation is more stimulating than the horizontal bars. Values of COP area and VOS in the horizontal bar and vertical optokinetic stimulations of the BRU were higher in elderly individuals with a history of instability and falls in the past year than in healthy individuals.^11^

In the case of inaccurate visual information (i.e., optokinetic stimuli), the postural control system must decide which sensorial input is the most appropriate to maintain orientation and postural stability.^4^ Under the test Conditions 6–8 (optokinetic stimuli), a greater body sway increase was observed in older adults with chronic vestibular disorders, which could be explained by an apparent preference of patients to accept any visual information, even if inappropriate, as correct. In these conditions, elderly patients with vestibular disorders may have difficulty selecting the most accurate sensory inputs, such as the somesthetic information.

In Condition 9 (horizontal visual–vestibular interaction), the COP area in the three groups differed and VOS differed only in G2 and CG, which is consistent with the findings in other studies.^11^, ^30^ In Condition 10 (vertical visual–vestibular interaction), only G2 showed significantly higher values of COP area than CG. Hence, horizontal head movement may be more stimulating or may provide greater visual conflict in relation to the flexo-extension head movement.

The limitations of our study included the following: (1) the software used does not allow randomization of the conditions, which may prevent the influence of learning; all participants were assessed in a standardized sequence by BRU (i.e., from the first to the tenth condition), (2) staying in the orthostatic position from the fourth to the tenth condition without a resting interval because of the difficulty in positioning on the platform (the participants were instructed to ask for rest whenever necessary), (3) the available parameters of BRU static posturography provided only mean values of the COP area and VOS, which were measured in 60 s for each condition However, the direction (i.e., midlateral and anteroposterior) that presented greater body oscillation was not verified.

## Conclusion

In conclusion, this study showed that patients with chronic vestibular disorder with or without falls have a reduced LOS area. However, patients with vestibular disorder without falls presented a body sway similar to that of healthy elderly individuals. Postural control of elderly individuals with chronic vestibular disorder with falls is worse than healthy individuals, and individuals with chronic vestibular disorder without falls.

These findings also support that posturography could quantify and identify patients at risk of falling, considering that the incidence of falls increases with increased body oscillation in elderly individuals.

## Ethical approval

This study was approved by the Research Ethics Committee of the Federal University of São Paulo (UNIFESP), protocol number 1704/07.

## Funding

Authors acknowledge the Coordenação de Aperfeiçoamento de Pessoal de Nível Superior (CAPES) for the financial support in this research.

## Conflicts of interest

The authors declare no conflicts of interest.
